# Overexpression of a single ORF can extend chronological lifespan in yeast if retrograde signaling and stress response are stimulated

**DOI:** 10.1007/s10522-021-09924-z

**Published:** 2021-05-30

**Authors:** Elzbieta Pogoda, Hanna Tutaj, Adrian Pirog, Katarzyna Tomala, Ryszard Korona

**Affiliations:** Institute of Environmental Sciences, Jagiellonian University, Gronostajowa 7, 30–387, Cracow, Poland

**Keywords:** Chronological lifespan, Budding yeast, Gene overexpression, Stress reaction, Retrograde signaling

## Abstract

Systematic collections of single-gene deletions have been invaluable in uncovering determinants of lifespan in yeast. Overexpression of a single gene does not have such a clear outcome as cancellation of its function but it can lead to a variety of imbalances, deregulations and compensations, and some of them could be important for longevity. We report an experiment in which a genome-wide collection of strains overexpressing a single gene was assayed for chronological lifespan (CLS). Only one group of proteins, those locating to the inner membrane and matrix of mitochondria, tended to extend CLS when abundantly overproduced. We selected two such strains—one overexpressing Qcr7 of the respiratory complex III, the other overexpressing Mrps28 of the small mitoribosomal subunit—and analyzed their transcriptomes. The uncovered shifts in RNA abundance in the two strains were nearly identical and highly suggestive. They implied a distortion in the co-translational assembly of respiratory complexes followed by retrograde signaling to the nucleus. The consequent reprogramming of the entire cellular metabolism towards the resistance to stress resulted in an enhanced ability to persist in a non-proliferating state. Our results show that surveillance of the inner mitochondrial membrane integrity is of outstanding importance for the cell. They also demonstrate that overexpression of single genes could be used effectively to elucidate the mitochondrion-nucleus crosstalk.

**Supplementary Information:**

The online version contains supplementary material available at 10.1007/s10522-021-09924-z.

## Introduction

The budding yeast *Saccharomyces cerevisiae* has been exceptionally useful in classic genetics, recombinant technology and systems biology. Its status of a model eukaryotic organism indispensable for research in multiple areas of modern biology is unquestioned (Botstein and Fink, 2011). Its role in the study of longevity and senescence is also well recognized although this particular success may appear somewhat paradoxical. Unlike most metazoan cells, the budding yeast cells divide asymmetrically, the larger cell is regarded as older (mother) and the smaller—younger (a bud or daughter). The mother cell can produce only a limited number of buds and the final count of them is its replicative lifespan (RLS), a measure that has no good analog in metazoans. Another measure of longevity, chronological lifespan (CLS), is the period between the origin and demise of a particular cell. It appears universal but does not mean the same for the yeast and metazoan cells. A metazoan cell can adopt a non-dividing state even if nutrients are available in its exterior while every yeast cell would grow and divide if provided with required materials. Therefore, any study of CLS in yeast is necessarily done under conditions testing not just the ability to persist alive but also to withstand starvation. It is thus remarkable that despite these incongruences the research on yeast RLS and CLS turned out so helpful in identifying the absolutely critical pathways of signaling and metabolism in ageing *metazoan* cells (Fontana et al., 2010). Apparently, a model does not need to be perfectly fitting as long as it is superbly tractable.

One particular advantage of the budding yeast is the availability of systematic collections of mutants in which every known gene is altered in a defined way. Exact deletions of a single open reading frame are especially useful because the effect of complete gene inactivation is often most informative about gene function. Therefore, even the laborious assays of RLS were applied to an entire collection of individual yeast gene deletions (McCormick et al., 2015). CLS can be assayed much more expediently, either with single strains or mixtures of them (Campos et al., 2018; Fabrizio et al., 2010; Garay et al., 2014; Gresham et al., 2011; Klosinska et al., 2011; Marek and Korona, 2013; Matecic et al., 2010; Powers et al., 2006). These systematic screens recapitulated insights gained in earlier and more narrow studies and added several new ones. They were especially successful in uncovering molecular mechanisms responsible for extending CLS under DR (dietary restriction) which is an arrangement adopted also in the present study. Poor nutritional conditions, signaled chiefly through the TOR and RAS/PKA pathways, lead to the activation of a cell-maintenance program which includes downregulation of biosynthesis, arrest of cell-cycle, intensification of respiration and other mitochondrial functions, development of stress resistance and expansion of autophagy. This catalog includes only broad functional categories. Each of them contains dozens of genes that commonly have metazoan homologs involved in extending lifespan of post-mitotic cells (A McCormick and K Kennedy, 2012; Campos and DeLuna, 2019; Kapahi et al., 2017).

Gene overexpression is applied less frequently than gene inactivation. It is also more difficult to interpret. It can result in intensification of functions typical for the protein product of an overexpressed gene but also in excessive sequestration of its partners, competition for subunits shared with other proteins, disruption of stoichiometric complexes, promiscuous interactions with proteins or nucleic acids, the toxicity of misfolded aggregates and other effects (Albrecht et al., 2018; Moriya, 2015; Prelich, 2012). The Saccharomyces Genome Database lists 1,270 ORFs exhibiting any effects on CLS when manipulated. Of those, only 27 relate to overexpression. As a group, the 27 ORFs are enriched in genes relating to the categories of “oxidation–reduction process” or “reactive oxygen species metabolic process” and it appears unsurprising that disruption of these elements of metabolism can affect CLS, usually negatively. Beside genes from those categories, the histone deacetylase coding *SIR2* has been reported to increase CLS while overexpression of its homolog *HST3* to decrease it which might be difficult to interpret especially that (de)acetylation of histones is highly pleiotropic (Orozco 2013). These insights appear modest, compared to those obtained by inactivation of genes, they were gathered after relatively few and rather narrowly targeted studies. Indeed, an attempt to uncover longevity genes thorough overexpression applied on a genomic scale has not been made yet.

In the present study, we use a collection of yeast strains each containing a multi-copy plasmid with one yeast ORF cloned after a promoter which can be induced to overexpression with galactose. The collection was created primarily to facilitate harvesting of single yeast proteins but it has been since used to study the effect of overexpression on some fitness components such as growth rate, competitive ability and genome stability (Biesiadecka et al., 2020; Gelperin et al., 2005; Tomala and Korona, 2013; Tutaj et al., 2019; Vavouri et al., 2009). Here we propagated large cultures containing complete pools of strains under conditions inciting strong overexpression and then transferred them to conditions of dietary restriction. We sampled the starving cultures periodically and amplified DNA of them to find which strains descended or ascended among survivors and thus learn about their CLS. There are two important features of this experiment that result from inherent traits of the applied collection. First, as glucose had to be excluded, it was raffinose that served as the main source of organic carbon at the phase of growth preceding starvation. Raffinose is poorly fermentable and allows for de-repression of respiration (Randez-Gil et al., 1998). Therefore, there was no abrupt termination of fermentation and adoption of oxidative metabolism at the end of growth which is typical for most studies of CLS in yeast. Second, unlike some collections that contain strains marked individually with easy to amplify tags located on chromosomes, our collection was composed of one untagged yeast host and a common core plasmid with individual ORFs cloned into it, only the latter defined strain identity. Numerous strains did not respond to simultaneous PCR amplification strongly enough to detect changes in their frequency. However, the obtained sample of amplified genes was sufficiently large and unbiased to reveal that an extension of CLS through overexpression is possible but restricted to genes coding for proteins localizing to the matrix and inner membrane of mitochondria. Subsequent analyses of transcriptomes of two different longliving strains suggested that an artificially engineered overabundance of these proteins was perceived as stress and incited a broad metabolic reaction leading to enhanced survival.

## Materials and methods

### Media, strains and plasmids

Standard synthetic complete medium (SC) with three different carbon sources was used. SC dropped for uracil and with 2% glucose served to propagate strains while the *P*_*GAL1*_ promoter was repressed. SC minus uracil with 2% raffinose served to de-repress the promoter. The latter medium with 2% galactose added was used to induce high expression of the cloned genes. Because the applied here strain of yeast, Y258, was unable to metabolize galactose, this sugar served only as an inductor while raffinose was a source of carbon (Gelperin et al., 2005). The MORF collection consists of 5188 single yeast ORFs fused after *P*_*GAL1*_ and followed by an affinity tag, His6-HA-ZZ, cloned into a 2-micron plasmid with the *URA3* marker and hosted by a haploid yeast strain Y258, *MATa pep4-3 his4-580 ura3-52 leu2-3* (Gelperin et al., 2005). We used a modified version of this collection that had been created in our earlier work (Tutaj et al., 2019). It was originally used to screen for loss of heterozygosity at the *CAN1/can1* locus but it also contained a gene conferring resistance to geneticin (*kan*) which helped maintain single and mixed strains free from external contaminations. The diploid Y258 host strain genotype was: *MATa/MAT pep4-3/pep4-3 his4-580/his4-580 ura3-52/ura3-52 leu2-3/leu2-3 CAN1/can1, MET6/met6::kanMX4.* It was propagated in the same media as listed above supplemented with 200 mg/l of geneticin (G418). A mixture of individual host strains was prepared to initiate a starvation experiment as described below.

### Starvation experiment

Titration plates with 200 µl of fresh SC with glucose were inoculated with frozen samples of individual diploid overexpression strains and brought to the stationary phase. Aliquots of 100 µl of every strain were combined to form a large mixed culture. Of that, 40 ml samples were transferred to 125 ml Erlenmeyer flat base flasks covered by DuoCap® caps with 0.22 µm membranes enabling ventilation. Eight such flasks were incubated for 24 h at 30 °C with shaking 200 rpm, then 4 ml inocula were transferred into 36 ml aliquots of fresh medium containing raffinose. Thus, the initial mixture contained about 6000 cells of every strain. The cultures were grown for 48 h at 30 °C/200 rpm. Of those, 5% inocula were transferred into aliquots totaling 40 ml, containing both raffinose and galactose. These cultures were grown for 24 h at 30 °C/200 rpm.

To initiate starvation, 4 ml samples of each culture were spun down and then suspended in 40 ml of media which did not support cell proliferation. Four samples were suspended in plain water (H treatment) and four in water supplemented with 2% galactose (G treatment). The starving cultures were kept at 30 °C/200 rpm, aerated via DuoCap® covers, evaporation was compensated by adding deionized water whenever visibly needed.

Immediately after the dilution and then after every 7 days, from day 0 to 28, 0.5 ml samples of the starving cultures were taken, spread on agar plates and incubated at 30 °C for 48 h. The plates contained 2% glucose, standard yeast nitrogen base, required amino acids, but not uracil, 0.2% peptone and 0.1% yeast extract. The last two compounds were included to help restarting metabolism in starved cells while the bulk of medium (SC lacking uracil) ensured high yields of cells hosting plasmids. Resultant cell lawns were washed with liquid SC lacking uracil, supplemented with 15% glycerol and deep frozen.

### DNA extraction, amplification and mapping

Samples collected over the whole experiment were simultaneously thawed and total DNA was isolated by cell lysis in lithium acetate-SDS solution and subsequent precipitation of DNA with ethanol (Lõoke et al., 2011). The cloned ORFs were amplified from flanking plasmid regions with the forward primer (5’ATT TGT TGT CCA CGG CCG AT) anchored in *P*_*GAL1*_ 75 bp upstream from the AUG codon in the yeast ORF sequence. Reverse primer (5’ACC TCT ATA CTT TAA CGT CAA GGA) started 75 bp downstream from the STOP codon of the ORF. Amplification was carried out with a high fidelity thermostable polymerase (Thermo Scientific Phusion Hot Start II High-Fidelity DNA Polymerase) from 1000 to 2000 ng total DNA concentration in 20 µl reactions with each primer at a final concentration of 0.5 µM. Elongation step was extended to 5 min to allow amplifications of the longest yeast ORFs. PCR products were inspected on a 1.5% agarose gel to confirm generation of a smear indicating a mixture of PCR products ranging from 500 bp to 14 kb. Excess primer and PCR reagents were removed using magnetic beads (Agencourt AMPure XP – PCR Purification, Beckman Coulter). DNA libraries were then prepared with a Nextera kit (MiSeq for Illumina).

DNA sequence reads were subject to a Cutadapt analysis in order to remove sequences flanking the searched ORF and for quality filtering (Martin, 2011). Next, reads were aligned with Bowtie2 (http://bowtie-bio.sourceforge.net/bowtie2/index.shtml) with options very-sensitive and -q30 (Langmead and Salzberg, 2012). Genomic sequences of all ORFs from the MORF collection were used as a reference set (S288C, R64.2.1). Output BAM alignment files were sorted and indexed with samtools (version: 0.1.19). Numbers of reads aligned to ORFs were obtained with the samtools idxstats command (Li et al., 2009).

Reads were counted for every strain in each of the four replications at each of the four time points of sampling independently for the H and G environments. Regressions of frequency over time were calculated for all strains, those with a total number of reads lower than 160 per environment were discarded from further analyses.

### Assays of ATP, ROS and cell viability

After comparing relative CLS of the starved strains, see Results, three groups of strains were defined and compared in these assays. The L (long-lived) group consisted of strains that increased in frequency in both the H and G environments except for those which occurred to be unsuitable for the planned assays, mostly because of forming cell aggregates. The final number of the tested L strains was 77. The S (short-lived) strains were recruited from among those which tended to decrease in frequency in both H and G and did it at the highest rate. The final count of strains qualified for these assays was 66. There were also 13 M (medium) strains which were selected as those closest to not changing in frequency over the course of starvation in both H and G.

The L, S and M strains were passed through propagation in SC with glucose, raffinose and raffinose plus galactose in the same way as when being prepared for starvation but as individual 200 µl cultures (agitated at 1000 rpm) instead of one common culture (see above). Incubation of the raffinose plus galactose cultures was terminated after 24 h, at the same moment as the initiation of starvation and then all of the analyses were carried out.

To measure the level of ATP, we used a CellTiter-Glo Luminescent Cell Viability Assay kit (Promega, G7570) following the manufacturer’s instructions. The strains have been equalized to a similar value of OD (0.07) and mixed in a 1:1 ratio with the reagent on multiwell plates and incubated for 10 min at room temperature. After the incubation, luminescence was measured with a Tecan reader and the reads were divided by the number of cells.

The level of ROS and cell viability was determined with flow cytometry using a 488 nm blue laser for excitation (CytoFLEX S, Beckman Coulter). Prior to analysis samples of 10^6^ cells were treated with CellROX® Green Reagent (Invitrogen) at a final concentration of 5 μM plus 5 μl/ml propidium iodide (Sigma-Aldrich). Plates were incubated for 30 min at 30 °C in the dark and the individual cell fluorescence was measured under both PE and FITC filters. Raw reads were then processed as described elsewhere (McBee et al., 2017). First, all single yeast cells with intact cell walls were gated. Then autofluorescence was taken into account by subtracting particles which showed fluorescent signals equal to control cells not stained with CellROX®Green. As the L, M and S groups differed in the size of cells (the differences were moderate, but L tended to be smallest while S largest), the size of non-stained control cells was selected to match that of each of the three compared groups. These “net” readings were then used to calculate a ratio of median fluorescence over mean cell size for every individual strain to avoid a possible bias resulting from the inequality of cell sizes. For each strain, three independently prepared samples were assayed and calibrated in the described above way and then averaged before being used in plots and statistical tests.

### RNA extraction and transcriptome analysis

Cultures were prepared as for the ATP/ROS assays except that aliquots were 5 ml. Tree replicas of each analyzed strain were independently propagated and then treated as follows. Total RNA was extracted with RiboPure™ RNA Purification Kit. Libraries preparation and PE 150 sequencing was carried out in Novagene. About 20 mln read pairs were obtained per sample. Quality control of the reads was performed with fastQC v0.11.9 (Andrew, 2010). Reads were then aligned to Ensembl release 100 *Saccharomyces cerevisiae* genome with Hisat2 v2.1.0 (Kim et al., 2015). Resulting alignment files were sorted and indexed with samtools (1.9). Transcript quantification was performed with cuffquant/cuffnorm v2.2.1 (Trapnell et al., 2012). Exact commands are given in Supplementary Methods. Gene count data normalization and differential expression analysis were performed in the EdgeR exact test (Robinson et al., 2010).

## Results

### Overexpression of single ORFs can affect chronological lifespan

Our goal was to compare the chronological lifespan (CLS) of multiple yeast strains kept in parallel in a single culture. Every strain consisted of the same yeast host (Y258) and a plasmid with a single yeast ORF inserted after a galactose-inducible promoter. We prepared large cultures of such cells in which particular plasmids were roughly evenly represented and the promoters fully induced (see Materials and methods). Two independent assays of CLS were then initiated: the cells were washed and suspended in either pure water, environment H, or water with galactose added, environment G. (Y258 does not catabolize galactose and therefore the sugar supposedly served only as a transcription inductor.) Samples of H and G were periodically taken, overlaid on plates and allowed to grow; the resulting cells were harvested and tested through NGS for the abundance of particular ORFs within a sample. Recorded over time, a tendency to increase or decrease in frequency would then serve to decide whether an overexpressed ORF had a positive or negative effect on longevity.

**Fig. 1:**
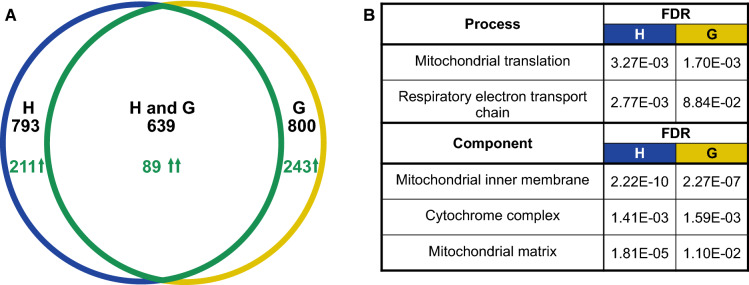
Impact of overexpression on the extension of relative CLS. (A) Overlap among strains which were amplified well through PCR from samples of the H cultures (water only) and G cultures (galactose added). The incidence of strains with an increased relative CLS is shown with arrows. (B) Enrichment in Gene Ontology categories among the top of the H and G lists of genes ranked from those of highest relative CLS (there were no enrichments with opposite rankings)

This approach, intended to reveal the impact of overexpression of every cloned gene, proved effective for only some of them because not all yielded sufficiently numerous products of amplification. Events of successful amplification were not random but tended to repeat strongly in all four replicate mass-cultures in H and also all four in G. Out of 6712 ORFs constituting the collection, 793 were amplified well in H and 800 in G. Of those, 639 overlapped which was much more than the number expected (94.52) if amplifications of samples coming from the two environments were independent (Fig. 1A). It suggested that further efforts would not result in many new ORFs added. Supplementary Table 1 provides lists of those genes, the criteria used to select them are described in the Materials and methods.

Our sample of successfully amplified genes is incomplete and therefore it is crucial to ask whether it is also biased. One known factor of the PCR efficiency is the distance between primers. Indeed, the well-amplifying genes tended to be somewhat underrepresented if long ORFs were considered (Supplementary Fig. 1A). But, it would be more important to know whether the well-amplified genes were of different significance for the cell than the rest. In our former work, we estimated effects on growth (as opposed to viability tested here) associated with amplification of every ORF in the same collection of strains (Tomala and Korona, 2013). Defined that way, the fitness of the successfully amplified sample was hardly distinguishable from the whole collection (Supplementary Fig. 1B). Thus, the choice of genes we had at our disposal was most likely dictated by molecular properties of DNA coding for them and not their biological significance. We acknowledge overlooking many individual effects but we consider it justifiable to use the remaining ones to investigate what functional *categories* of genes affect CLS when overexpressed.

### CLS is extended when elements of the mitochondrial interior are overexpressed

Using the four replicate assays, we first calculated an average slope of frequency change for every amplified gene in either the H or G environment. Figure 1A shows that there were only 89 strains with two positive average slopes (shown in Supplementary Table 1). These 89 well-surviving strains are listed and shortly described in Supplementary Table 2. To test for possible functional factors of CLS, we ranked all the well-amplified strains according to an average slope in either H or G environment beginning with either the best or worst surviving ones. A statistical test for overrepresentation of GO terms among top ranks (Eden et al., 2009) yielded similar results for both environments. There were no enrichments among the shortest living strains but the longest living ones were highly enriched in the Gene Ontology categories relating to the mitochondrial matrix and inner membrane. These results are summarized in Fig. 1B and fully reported in Supplementary Table 3.

### Long living strains tend to have higher ATP and lower ROS levels

**Fig. 2:**
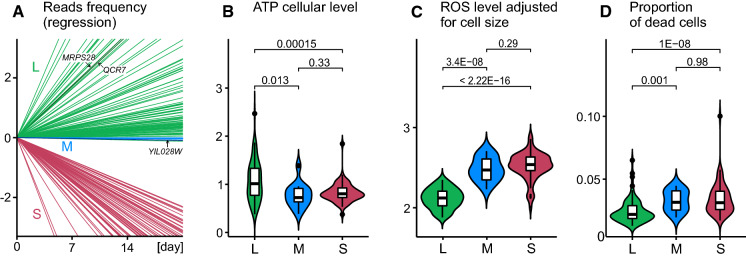
Cellular level of selected metabolites in L, M and S (long, medium and short-living strains). (A) The performance of L, M and S strains is shown as regression slopes of frequencies among alive cells over time in aging mixtures of all strains. Frequencies were subjected to angular transformation and therefore the Y-axis is scaled in degrees (Sokal and Rohlf, 1995). Intercepts were subtracted to pin all lines at a common starting point. Every line represents an average of eight slopes, four from H and four from G treatments. Individual labels denote the three strains that were used in subsequent transcriptome analyses. (B-D) The Y-axis for ATP and ROS is linear with arbitrary units (see details in Methods). Type I errors in pairwise comparisons are shown atop brackets

The above GO analyses employed internal comparisons within the sample of overexpressing strains. We continued this approach of internal comparisons when scrutinizing physiological parameters of the overexpressing strains. We first defined three groups of strains. The L (long-lived) group were those which increased in proportion under both the H and G conditions, the S (short-lived) ones were represented by those which decreased most markedly under both conditions, and M (medium) ones were those which tended to stay unchanged in proportion over the starvation period. In such a design, groups of different performance constitute reciprocal “controls” for each other. Figure 2A compares the relative performance of L, M and S strains shown as increasing or decreasing regression lines of their proportions among alive cells in the aging population. (ORF names are listed in Supplementary Table 4.)

Having these three groups established, we began our assays by harvesting cells at the end of growth under the overexpression regime, that is, cells representing the same metabolic state as those entering starvation in the described above longevity assays. We measured the content of ATP, whole-cell content of ROS (reactive oxygen species) and proportion of alive vs. dead cells. Figures 2B-D show that of all possible differences one was consistent, the L strains differed from the two remaining groups. Specifically, they had a higher level of ATP, lower level of ROS and lower proportion of dead cells. The S and M were largely similar to each other confirming the results of the GO analysis, that is, only the longest living strains constituted a group distinct from other strains.

### mRNA profiles of long-living strains point to retrograde signaling

Two strains, overexpressing either *QRC7* or *MRPS28*, were selected for an analysis of their transcriptomes. The first of them codes for a protein that is imported through the outer membrane and then inserted into the inner membrane of a mitochondrion as a member of the electron transport chain Complex III. The second gene codes for a protein imported into the mitochondrial matrix and incorporated into the small subunit of a mitochondrial ribosome. We also selected a strain overexpressing YIL028W which is (most likely) a spurious ORF, non-overlapping with other ORFs and flanked by about one thousand base pairs of non-coding DNA on both sides. We considered it the best accessible control for *QRC7* and *MRPS28* transcriptomes. The control strain had the same core plasmid and therefore benefited from nutritionally important *URA3*. The burden was also the same, a multicopy 2µ DNA molecule and intense transcription concentrated on *P*_*GAL1*_ (indeed visible in the reported below Results). Thus, the experimental and control strains differed in only one aspect, the informational significance of the overproduced mRNA and protein. Note, that CLS of *QRC7* or *MRPS28* was typical for the reported earlier L strains while that of YIL028W was typical for the M strains (Fig. 2A).

**Fig. 3:**
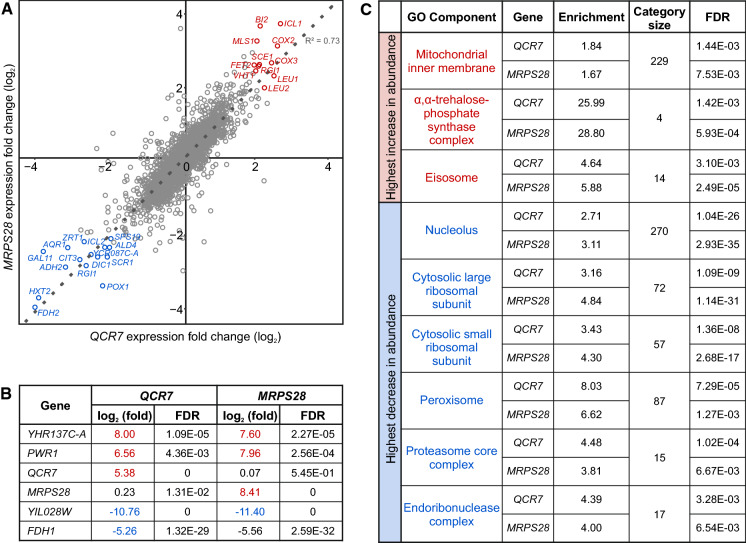
Transcriptional response to overexpression of two selected genes. (A) Correlation between upward and downward shifts in RNA abundance in the two test strains, both compared to the common control. (B) Increases or decreases higher than 16 times and hence not fitting graph A. (C) GO categories enriched at the tops of lists with RNAs ranked from either the most increased or decreased abundances

The three strains were cultured in the same way as the earlier described gene mixtures but instead of entering starvation, they were subjected to RNA extraction. Figure 3 compares the *QRC7* and *MRPS28* transcriptomes in terms of their deviations from the control. An overall pattern is striking: there was a strong correlation in the direction and magnitude of shifts in expression of all (about 6,000) recognizable mRNAs and other short RNAs. Furthermore, genes showing fourfold up- or downregulation were nearly the same for the two transcriptomes, they are labeled individually in Figs. 3A and 3B. (Fig. 3B shows also that transcripts of *QRC7, MRPS8* and YIL028W were especially abundant in strains carrying respective overexpression plasmids, as expected.) To learn about genes with effects smaller than those named individually in Figs. 3 A and B, we ranked all mRNAs beginning with those most upregulated or those most downregulated. We then tested for enrichments in the GO categories at both ends of the spectrum. Full results are provided in Supplementary Table 5, a short list of overrepresented cellular components is displayed in Fig. 3C.

Genes highlighted in Fig. 3 belong to relatively few but substantially different functional categories. To begin, among about a dozen RNAs showing at least a fourfold increase under both *QRC7* and *MRPS28* overexpression, there were as many as four elements coded on the mitochondrial genome: *COX2*, *COX3*, *BI2* and *SCEI*. We assume that their overabundance resulted from impediment(s) in the assembly of the electron transport chain complexes in the inner membrane of mitochondria, we explain this assertion in more detail in Discussion. Here, we provide a short overview of results obtained through our analyses of the abundance of RNA species. Specifically, we propose that the overabundance of several elements coded by mtDNA reveals a disturbance that led to a retrograde signal sent from the mitochondria and perceived in the nucleus. A number of the most pronounced shifts in the level of messenger RNA could be explained as elements of a programmed modification of the cellular metabolism required to minimize damage and prepare for stress. Namely, to lower the danger of leakage of electrons from the malfunctioning chain, the TCA cycle as a source of NADH was suppressed (down: citrate synthase *CIT3*). Other sources of NADH were also dampened (down: formate dehydrogenases *FDH1, FDH2*, alcohol dehydrogenase II *ADH2*). An impairment of the TCA cycle has to be compensated by activation of the glyoxylate cycle (Jazwinski, 2013). There were unmistakable signs of that in our data (up: isocitrate lyase *ICL1*, malate synthase *MLS1*, isopropylmalate dehydrogenase *LEU1*, β-isopropylmalate dehydrogenase *LEU2*). The glyoxylate cycle provides simple building blocks for the synthesis of carbohydrates, including trehalose which is a standard storage/protective material in the yeast cell (up: trehalose-phosphate synthase complex). Another change that likely helped to prolong CLS was to spare lipids by decreasing the rate of importing them to peroxisomes (down: transmembrane fatty acid transporter coding *PXA1*) and oxidizing them therein (down: *DCI1*, *ECI1*, *FOX2*, *POX1*, *POT1*, *PEX11*). There was also a decrease in protein turnover, that is, their synthesis (down: elements of the nucleolus, cytosolic ribosomes) and degradation (down: proteasome core complex). Smaller translational apparatus meant also that nuclear transcription (typically dominated by ribosomal protein genes) was less intense (down: RNA pol II coactivator *GAL11*).

## Discussion

A simplified expectation in overexpression studies would be that genes that are known to shorten/lengthen lifespan when present at native levels have even stronger effects when overexpressed. We detected nearly one hundred ORFs that extended CLS when overexpressed but we did not see among them those associated with the metabolic processes or signaling pathways known to aid cell longevity. We thus conclude that none of the complex functions needed to prepare for persistence under non-proliferation could be effectively boosted by overexpressing a single gene. Products of the genes extending lifespan localized overwhelmingly to just one cellular region: the inner membrane and matrix of mitochondria. But, the extra protein particles were unnecessary, as native expression was present, and they rather tended to distort than help. As we argue below, this artificially created disturbances triggered a programmed stress response which typically serves to counter similar, if less acute, naturally occurring metabolic problems.

One indication that the long-living strains were indeed prepared to resist starvation is the fact that they had higher levels of ATP and lower levels of ROS when compared either with the short-living or medium-living strains. While the positive longevity effect of high energy reserves accessible as ATP appears understandable, the meaning of the observed low ROS content may require some consideration. First of all, the level of ROS could actually be elevated if the overexpressed protein damaged the inner mitochondrial membrane. Furthermore, the relation between the cellular level of ROS and lifespan is not entirely clear. ROS is an agent of damage but also a possible signal. It has been proposed that an increased mtROS production constitutes a signal leading to CLS extension via TORC1 mediated silencing of subtelomeric regions (Pan et al., 2011; Schroeder et al., 2013). More generally, it has been often postulated that a rising level of ROS itself is a signal of mild stress enabling the cell to prepare for subsequent perturbation, a phenomenon termed “mitohormesis” (Yun and Finkel, 2014). Our data do not support this line of reasoning. The long-living strains were better equipped with energy and not burdened with potentially damaging ROS. Was the latter neutralized more efficiently or produced less intensely?

A likely answer is that CLS was extended in strains that produced less ROS and were able to redirect resources from growth to stress resistance. This explanation is suggested by an analysis of transcriptomes of two long-living overexpression strains, *QCR7* and *MRPS28*. The strikingly correlated shifts in the abundance of their RNAs (Fig. 3) are likely explained by the fact that the two proteins coded by them are tied not only by localization but also cellular function. Both are imported from the cytosol and inserted, respectively, to the Complex III of the electron transport chain (tightly embedded into the inner membrane) and the small subunit of the mitochondrial ribosome (in the matrix but probably mostly tethered to the inner membrane). Yeast mitochondria synthesize only seven proteins, six of them are parts of electron transporting complexes. An emerging polypeptide folds and inserts into the inner membrane co-translationally. This new protein is released from its “parental” mRNA/ribosome pair only after it is assembled with other components of a relevant respiratory complex (Herrmann et al., 2013). This control mechanism prevents the proliferation of incomplete and thus unsafe complexes. It also suggests an explanation of our results. The assemblage of respiratory complexes from proteins translated in situ and those imported from the cytosol requires precise coordination. Apparently, both disturbed translation (caused by excessive Mrps28) and abnormal stoichiometry of protein complexes (excessive Qcr7) resulted in the same general failure which led to a uniform reaction of an entire cell.

The postulated failure was flagged by the increased abundance of the same few transcripts encoded on mtDNA in both *QCR7* and *MRPS28* overexpressing strains. These were two mRNAs, *COX2* and *COX3* (members of Complex IV). *AI1* and *AI2* (reverse transcriptases required for splicing of pre-mRNA of *COX1*, member of Complex IV) as well as *AI4* (endonuclease *I-SCEII*), all encoded by introns within *COX1*, were also overrepresented. Highly abundant in both overexpressing strains were transcripts of the DNA endonuclease *I-SCEI* (encoded on an intron within mitochondrial rRNA) and mitochondrial mRNA maturase *BI2* (encoded by both exon and intron sequences of partially processed mRNA of cytochrome b, the central subunit of Complex III). Accumulation of these particular elements most likely resulted from impediments in the assemblage of respiratory complexes (Fontanesi, 2013). An alternative interpretation would be that an enhanced level of those few RNAs coded by mtDNA indicated an enhanced rate of production of respiratory complexes. It is unlikely because there were no signs of intensified expression of other elements of the respiratory complexes or mitochondrial ribosomal proteins. Especially the latter would be easily detected as enrichment in relevant Gene Ontology terms among the most upregulated genes. However, downregulation of genes coding for elements of mitochondrial ribosomes was not visible either. It appears that neither problems with the import and assemblage of proteins synthesized in the cytosol nor those met during the mitochondrial translation but rather the inability to finish the whole process of assembling respiratory complexes in a coordinated way constituted the critical failure. How this failure was communicated to the nucleus is not clear, as it will be discussed below.

It is clear, however, that nuclear transcription reacted in largely the same way regardless of whether *QCR7* or *MRPS28* was overexpressed. Considering changes in the abundance of individual transcripts, it appears that the TCA cycle was cooled down while gluconeogenesis heated up. As for the GO categories, the easiest to see was the decrease in transcripts relating to the assembly of ribosomal units in the nucleolus and thus also the load of ribosomes in the cytosol. Components of the proteasome and peroxisome were also less abundant. There were only a few enriched categories, they covered the mentioned genes coded by the mtDNA. An interesting, and much telling, exception was the high expression of the trehalose synthase complex. In sum, these shifts amount to a clear sign of downregulation of the growth metabolism and preparation for stress and/or starvation. Such adjustments are known to result from decreased growth-stimulating signaling through TOR1 and PKA due to worsening environmental conditions and are among the best-recognized factors extending CLS (Kapahi et al., 2017). Here, we sampled cells still growing, although on poorly fermentable substrates, and therefore with likely upregulated oxidative metabolism (Guaragnella et al., 2013). There was no abrupt change in the quality of the environment or switch to the stationary state metabolism. It seems that, on top of the presumed adaptation to these rather unfavorable nutritional conditions (shared by *QCR7*, *MRPS28* and YIL028W), it was overexpression of only *QCR7* and *MRPS28* that led to a further re-profiling of transcriptome and the resulting calming of energetically expensive growth factors in favor of preparing for stress/starvation.

As for the question how a signal of mitochondrial malfunction can be perceived in a nucleus, there are several possible answers. One candidate way of communication is the RTG pathway (Parikh et al., 1987). RTG signals malfunctioning of mitochondria caused by a damage/loss of mtDNA. Our strains did not lack mtDNA and were able to grow not only on poor sugars but also on strictly non-fermentable compounds. However, proper expression of the mitochondrial genome is crucial for its maintenance as the processing of mitochondrial RNA and DNA is interconnected (Lipinski et al., 2010). Therefore, also in our strains, impaired metabolism of RNA/DNA could have occurred and initiated a signal of problems in the functioning of mitochondria. A link between the RTG signaling and extended RLS is well known (Kirchman et al., 1999). Another important constituent of mitochondria is their translation apparatus. Defective assembling of mitoribosomes has been linked to slower cytoplasmic translation and an increased RLS (Delaney et al., 2013). Similarly, impaired regulation of mitochondrial translation rate or accuracy can trigger stress reaction, retrograde signaling, and RLS or CLS extension (Caballero et al., 2011; Suhm et al., 2018; Suhm and Ott, 2017). Not only structural but also metabolic features of mitochondria in our strains with an increased CLS were specific. The TCA cycle was repressed. Moreover, nitrogen was scarce under the conditions of starvation. It has been found that the RTG pathway responds both to mitochondrial dysfunction and to nitrogen scarcity (Liu and Butow, 2006). RTG signaling is connected with that of the TOR1 pathway, which was likely responsible for the observed broad reprogramming of transcription and the resulting RLS extension (Borghouts et al., 2004; Giannattasio et al., 2005; Guaragnella et al., 2018; Jazwinski, 2013). Thus, both DNA and RNA of the mitochondrion, its translational apparatus, and its metabolism could have been affected in a way that initiated a signal transmitted to the nucleus. Our results do not say which specific molecular mechanism, or perhaps multiple mechanisms, were involved.

However, we may point to some modes of retrograde communication which are rather poor candidates to be involved here. It has been suggested that mitochondrial malfunctioning could be signaled by a change in the concentration of small molecules. In particular, a decrease in the level of ATP and/or increase in the level of ROS (Torelli et al., 2014; Zhang et al., 2013). However, our long-living strains had high levels of ATP while low of ROS and, as we have already explained, we tend to see this finding as an indication that the cellular metabolism was well controlled, not disturbed. Other rather unlikely ways of communication are those responsible for signaling disturbances in proteostasis. They are activated when precursors of mitochondrial protein accumulate on the outer surface of mitochondria or within their transportation channels (Weidberg and Amon, 2018; Wrobel et al., 2015). This is because we have overexpressed many different mitochondrial proteins, which likely misfolded and aggregated, but an extension of CLS was visible only in the case of those targeting respiratory complexes and ribosomes. Yet another rather unlikely mechanism of signaling would be that invoking small peptides resulting from proteolysis of the respiratory complexes. Excessive or damaged proteins of this kind would produce specific peptides which would constitute an unmistakable signal of problems in the inner membrane (Arnold et al., 2006). In our case, only overexpressed members of the chain would spawn such peptides in large amounts leading to the observed stress response. But, we see it for other mitochondrial proteins as well, for example, those constituting ribosomes. The research on retrograde signaling is far from completion but progress is evident (Andréasson et al., 2019; Guaragnella et al., 2018). We hope that our approach to stimulate mitochondrial stress, and the insights obtained by applying it, will help in these efforts.

A cautionary note should be added that our experimental system might have been especially effective in detecting some drastic but not subtle effects of overexpression. The collection of strains was devised specifically to produce mRNA and initiate translation at a very high and uniform level (due to the activity of *P*_*GAL1*_ and the coded by it upstream region of mRNA) and then to protect polypeptides from degradation (due to deletion of *PEP4*). The normal interplay between transcription, translation, post-translational modification and degradation of proteins is much more nuanced and often varies profoundly between individual genes (Beyer et al., 2004; Liu et al., 2016). It is possible that overexpressing to levels closer to native ones and allowing for adequate post-transcriptional regulation would lead to boosting or calming some metabolic or signaling activities in such a way that CLS would increase. Therefore, our study is far from answering all questions about the relationship between enhanced gene expression and altered lifespan of the yeast cells.

## Supplementary Information

Below is the link to the electronic supplementary material.

Supplementary file1 (DOCX 69 KB)

Supplementary file2 (XLSX 51 KB)

Supplementary file3 (XLSX 19 KB)

Supplementary file4 (XLSX 29 KB)

Supplementary file5 (XLSX 32 KB)

Supplementary file6 (XLSX 18 KB)

Supplementary file7 (DOCX 13 KB)

## Data Availability

On-line supplementary materials.

## Code availability

Non applicable.
