# Supplementary material for: Overexpression of a single ORF can extend chronological lifespan in yeast if retrograde signaling and stress response are stimulated
Source: Biogerontology. 2021 May 30;22(4):415–27. doi: 10.1007/s10522-021-09924-z (PMC8266792; doi:10.1007/s10522-021-09924-z)
Supplement: Supplementary file 1 — Supplementary file1 (DOCX 69 KB) [file 10522_2021_9924_MOESM1_ESM.docx]

**Supplementary Figure 1**


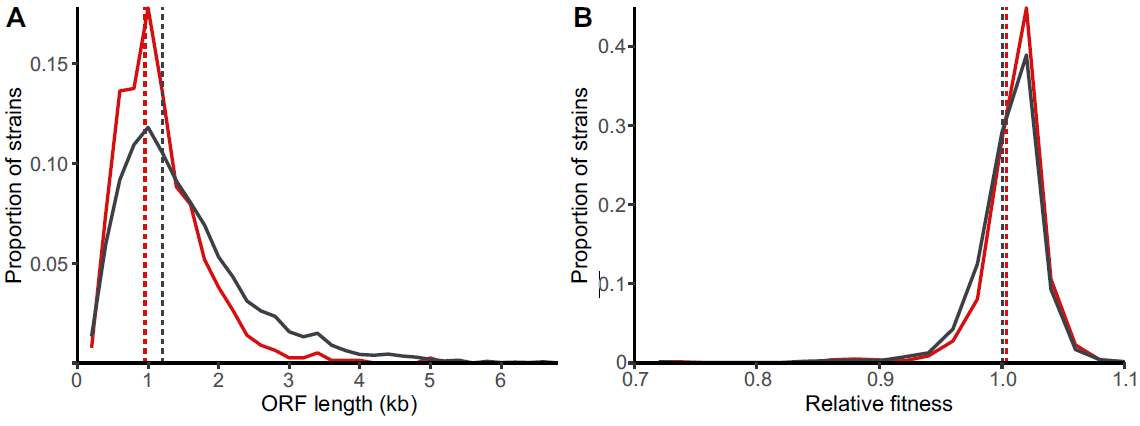


The sample of ORFs well amplified in PCR and thus used in this study (red) on the background of all ORFs (black). Lengths of overexpressed ORFs (A) and effects of overexpression on fitness as determined by another study (B) are compared.
