# Supplementary material for: Overexpression of a single ORF can extend chronological lifespan in yeast if retrograde signaling and stress response are stimulated
Source: Biogerontology. 2021 May 30;22(4):415–27. doi: 10.1007/s10522-021-09924-z (PMC8266792; doi:10.1007/s10522-021-09924-z)
Supplement: Supplementary file 7 — Supplementary file7 (DOCX 13 KB) [file 10522_2021_9924_MOESM7_ESM.docx]

**Supplementary methods**

RNA reads aligment commands

wget -O - "ftp://ftp.ensembl.org/pub/release-100/gtf/saccharomyces_cerevisiae/*.100.gtf.gz" | gunzip > scer.gtf

wget -O - "ftp://ftp.ensembl.org/pub/release-100/fasta/saccharomyces_cerevisiae/dna/*.toplevel.fa.gz" | gunzip > scer.fa

hisat2-build scer.fa scer

hisat2_extract_splice_sites.py scer.gtf > scer.splicesites

## RUN HISAT2

for name in $( ls *_1.fq.gz | cut -f1 -d "." | sed 's/_1//g' )

do

hisat2 -t --known-splicesite-infile scer.splicesites \

--dta-cufflinks -x scer \

-1 "$name"_1.fq.gz -2 "$name"_2.fq.gz -p 4 --summary-file "$name".txt --new-summary \

| samtools sort -@ 4 -O BAM -o file.bam

samtools index "$name".bam "$name".bam.bai

done

### CUFFQUANT

for name in $( ls *.bam | cut -f1 -d '.' )

do

cuffquant -v -p 4 --no-effective-length-correction -o "$name"/cuffquant \

scer.gtf "$name".bam

mv "$name"/cuffquant/abundances.cxb "$name"/cuffquant/"$name".cxb

done

### CUFFNORM

cuffnorm -p 4 -v -library-norm-method classic-fpkm -o cuffnorm-over \

scer.gtf $( ls */cuffquant/*.cxb | tr '\n' ' ' )
